# 250 years of hybridization between two biennial herb species without speciation

**DOI:** 10.1093/aobpla/plv081

**Published:** 2015-07-17

**Authors:** Andrew Matthews, Katie Emelianova, Abubakar A. Hatimy, Michael Chester, Jaume Pellicer, Khawaja Shafique Ahmad, Maité S. Guignard, Germinal Rouhan, Douglas E. Soltis, Pamela S. Soltis, Ilia J. Leitch, Andrew R. Leitch, Evgeny V. Mavrodiev, Richard J. A. Buggs

**Affiliations:** 1 School of Biological and Chemical Sciences, Queen Mary University of London, Mile End Road, London E1 4NS, UK; 2 Department of Biology, University of Florida, Gainesville, FL 32611, USA; 3 Department of Plant Science, University of Oxford, South Parks Road, Oxford OX1 3RB, UK; 4 Royal Botanic Gardens, Kew, Richmond, Surrey TW9 3DS, UK; 5 Lab of Plant Taxonomy, Department of Botany, University of Agriculture, Faisalabad 38040, Pakistan; 6 Museum national d’Histoire naturelle, UMR CNRS 7205, Herbier National, CP3916 rue Buffon, F-75231 Paris, France; 7 Florida Museum of Natural History, University of Florida, Gainesville, FL 32611, USA; 8 Present address: Division of Ecology and Evolution, Imperial College London, Silwood Park Campus, Ascot, UK

**Keywords:** Homoploid, hybridization, invasive, speciation, *Tragopogon*

## Abstract

Hybridization between plant species can generate novel morphological diversity and lead to speciation at homoploid or polyploid levels. Hybrids between biennial herbs *Tragopogon pratensis* and *T. porrifolius* have been studied in experimental and natural populations for over 250 years. Here we examine their current status in natural populations in southeast England. All hybrids found were diploid; they tended to grow taller and with more buds than their parental species; many showed partial fertility; a few showed evidence of backcrossing. However, we found no evidence to suggest that the hybrids are establishing as a new species, nor can we find literature documenting speciation of these hybrids elsewhere. This lack of speciation despite at least 250 years of hybridization contrasts with the fact that both parental species have formed new allopolyploid species through hybridization with another diploid, *T. dubius*. Understanding why hybrids often do not speciate, despite repeated opportunities, would enhance our understanding of both the evolutionary process and risk assessments of invasive species.

## Abstract (precis)

In 1759, Linnaeus convinced his followers that plants could hybridise by crossing flowers in the daisy family and producing intermediate offspring. These hybrids, between *Tragopogon pratensis* and *T. porrifolius*, exist naturally today in London, to all appearances the same. We find that most of the London hybrids are in their first generation, though we provide chromosomal evidence that one is a little older. These hybrids do not seem to have given rise to a new species, even though both parents have produced new hybrid species in the last century when crossed with *T. dubius*.

## Introduction

‘I obtained *Tragopogon hybridum* two years ago about autumn, in a small enclosure of the garden, where I had planted *Tragopogon pratense* and *Tragopogon porrifolius*, but the winter supervening destroyed the seeds. Early the following year, when *Tragopogon pratense* flowered, I rubbed off the pollen early in the morning, and at about eight in the morning I sprinkled the pistils with pollen from *Tragopogon porrifolius* and marked the calices with a thread bound around them. From these, towards autumn, I collected the mature seeds, and sowed them in a separate place, where they germinated, and in this year 1759, gave purple flowers with yellow bases, the seeds of which I now send.’ (pp. 126–127.)

Carolus Linnaeus (1760) *Disquistitio de Sexu Plantarum*, translation from Roberts (1929) *Plant Hybridisation before Mendel*, Princeton University Press, p. 22.

Since 1760, when Linnaeus published his *Disquistitio de Sexu Plantarum*, taxonomists have known that hybridization is an evolutionary source of novel morphological variation in plants (Linnaeus 1760; Roberts 1929; as Zirkle 1934, notes, the first artificial plant hybrids are credited to Thomas Fairchild of Hoxton in 1717, but his findings were not widely accepted). Even though hybrid novelties did not fit neatly with Darwin's later emphasis on gradualism and divergence in evolution (Darwin 1859), the evolutionary importance of hybridization has continued to be demonstrated and advocated by successive generations of biologists since Darwin (e.g. Lotsy 1916; Anderson and Stebbins 1954; Arnold 1992; Rieseberg *et al*. 2003) and is widely accepted today (Soltis and Soltis 2009; Abbott *et al*. 2013). Hybridization is now a well-attested mechanism for speciation, particularly when accompanied by genome doubling to produce allopolyploids (Rieseberg 1997; Mallet 2007; Soltis and Soltis 2009).

Linnaeus’ (1760) work was largely based upon crossing experiments between *Tragopogon pratensis* and *T. porrifolius*. These early experiments were repeated by Focke in Bremen, Germany (Focke 1890, 1897, 1907), by Winge in 1921 in Denmark (Winge 1938) and by Lotsy in the Netherlands (Lotsy 1927). Focke's and Winge's crosses yielded hybrids of similar morphology to Linnaeus', with purple outer ligules and yellow inner florets in the inflorescence, but Lotsy's showed a range of phenotypes not having a yellow centre to the inflorescence (Clausen 1966). Focke noted that some achenes produced by the hybrids germinated (Focke 1890) and that *T. pratensis* was normally the maternal parent of the hybrids (Focke 1897). The cross has also been repeated twice using North American accessions (Ownbey and McCollum 1953; Fahselt *et al*. 1976; Tate *et al*. 2009), derived from European parental accessions introduced to North America by settlers, yielding a range of inflorescence phenotypes in the *F*_1_, some having yellow central flowers, and some not. Several of the above studies harvested viable seeds from the *F*_1_ hybrids, and showed segregation of traits in *F*_2_ generations (Linnaeus 1760; Lotsy 1927; Winge 1938; Clausen 1966).

Hybrids between *T. porrifolius* and *T. pratensis* have been observed in the wild in Scandinavia for over 150 years. The PhD thesis of Carl Gosselman (1864) is reported to contain notes of this hybrid near Karlskrona in Sweden (Rouy 1890). Wilhelm Focke (1881, p. 222) reported that Johan Lange found spontaneous hybrids between *T. porrifolius* and *T. pratensis* on the Danish islands of Laaland and Funen: ‘the outer flowers brown-violet, the inner yellow’. In 1885, Knut Thedenius (1885) reported the hybrid in Stockholm. In 1890, Rouy named the hybrid *T.*×*mirabilis* and noted that it had been found by Gosselman (see above), and later by Foucard, Termonia and Maire in three locations in northern France (Rouy 1890). A population of *Tragopogon* diploids found in the Czech Republic were initially identified as hybrids between *T. porrifolius* and *T. pratensis* (Krahulec *et al*. 2005), but molecular investigations throw this identification into question (Malinska *et al*. 2011; Mavrodiev *et al*. 2013). Intriguingly, earlier than any of these reports, the 14th Volume of the *Flora Danica*, published in 1780, contains a colour plate (DCCXCVII) labelled as *T. porrifolius* in which the inflorescence has a yellow centre surrounded by purple outer ligules (Müller 1780); this may have been a hybrid found within a *T. porrifolius* population. Natural hybrids between *T. porrifolius* and *T. pratensis* have also been observed in North America since 1890 (Halsted 1890; Sherff 1911; Farwell 1930; Ownbey 1950; Clausen 1966; Novak *et al*. 1991).

Recent phylogenetic investigations of the genus *Tragopogon* confirm that *T. pratensis* and *T. porrifolius* are separate species, found in different phylogenetic clades. These investigations also suggest that purple-flowered European diploids (2*n* = 12) identified as *T. porrifolius* are polyphyletic with the most widespread lineage being the ‘salsify’ lineage (Mavrodiev *et al*. 2007). The yellow-flowered *T. pratensis* may also be non-monophyletic (e.g. Mavrodiev *et al*. 2012), but its most widespread lineage together with its sister species *T. minor* (or *T. pratensis* subsp. *minor*) consistently appears in the sub-clade Tragopogon. We cannot therefore be certain which lineages were involved in experimental crosses in the past, or which ones naturally hybridized, though the most widespread lineages would seem to be the most likely candidates. It seems probable that at least those hybrids with inflorescences appearing purple with a yellow centre have been repeatedly formed from the same parental lineages.

*Tragopogon* has become established as a model system for the study of hybridization, due to the discovery of various allopolyploid species (Ownbey 1950; Diaz De La Guardia and Blanca 2004; Mavrodiev *et al*. 2008*a*, *b*, 2015), documentation of further homoploid hybrids (reviewed in Buggs *et al*. 2008), a thorough phylogenetic framework (Mavrodiev *et al*. 2004, 2005), the recent resynthesis of hybrids and allotetraploids from their diploid progenitors (Tate *et al*. 2009) and transcriptome sequencing (Buggs *et al*. 2010). One intriguing feature of hybrid evolution in *Tragopogon* is a natural crossing ‘triangle’ among *T. dubius*, *T. porrifolius* and *T. pratensis* (all 2*n* = 12) within which natural allopolyploids (2*n* = 24) have formed repeatedly in the last 80 years from hybridizations involving *T. dubius*, but only homoploid hybrids have been recorded in nature between *T. porrifolius* and *T. pratensis*. In recent studies, much progress has been made on understanding the origin and rapid genome evolution of the two allopolyploids (*T. mirus* and *T. miscellus*) within this triangle (from *T. dubius*×*T. porrifolius* and *T. dubius*×*T. pratensis*, respectively) (reviewed in Soltis *et al*. 2012), but we know comparatively little about the homoploid hybrids between *T. porrifolius* and *T. pratensis*.

Both *T. pratensis* and *T. porrifolius* have been recorded in Britain since the 16th century to the present, *T. pratensis* being a native, and *T. porrifolius* considered to be a horticultural introduction (Gerard 1597; Stace and Crawley 2015). The oldest vouchered record of *T. porrifolius* in Britain is dated 1721, *Bobart Hortus Siccus Sectio* VIIA, p. 56 (OXF), collected from Sherard's garden at Eltham, London; *T. porrifolius* is also noted growing wild there (Dillenius 1732). Archaeobotanical records show *Tragopogon* sp. seed from Mid-Roman middens in York, dated between 150 and 200 AD (Hall and Kenward 1990). Hybrids have also been reported in Britain (Britton and Todd 1910; Ellis 1929; Clausen 1966; Burrow and Burrow 1978; Stace 2010). Druce curated *T. porrifolius*×*T. pratensis* specimens, including hybrids occurring naturally (F. Stratton, 1877; Dixon and Druce, 1907; Todd and Britton, 1910; H.E. Green, 1922; H. Wallis Kew, 1942, (OXF)) and hybrids produced by experimental crosses (C.E Britton, 1916, (OXF)).

This study aims to lay the foundations for the genetic and genomic study of *T. porrifolius*×*T. pratensis* hybrids, by identifying and characterizing natural populations. Here, we sampled six sites in southeast England reported to contain populations of *T. porrifolius*×*T. pratensis* hybrids, also sampling *T. porrifolius* and *T. pratensis*, if present at the sites. By analysing genome sizes, DNA sequences, morphology and seed fertility we explored the nature of the hybrids. Having confirmed their parentage, we asked: (i) Is there evidence for allopolyploid or homoploid hybrid speciation? (ii) Is there potential for gene flow between the two parental species?

## Methods

### Sampling

**Figure 1.**
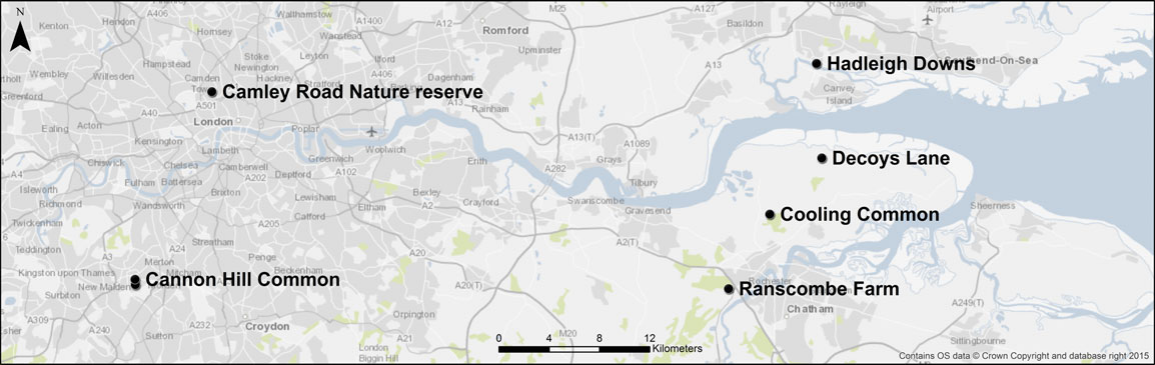
Location of the six populations sampled in southeast England, UK.

Sites in southeast England reported to contain putative *T. porrifolius*×*T. pratensis* hybrids were located by examination of botanical records and conversations with local botanists and county recorders for the Botanical Society of the British Isles. Six potential sites (Fig. 1) were visited between May and September 2011, with initial identification of *Tragopogon* species made using inflorescence morphology according to Stace (2010). At each site we aimed to collect equal numbers of plants of *T. porrifolius*, *T. pratensis* and putative hybrids, but this was rarely possible, and the collections made roughly reflected the overall frequency of each taxon at each site. Only plants with inflorescences were collected.

**Figure 2.**
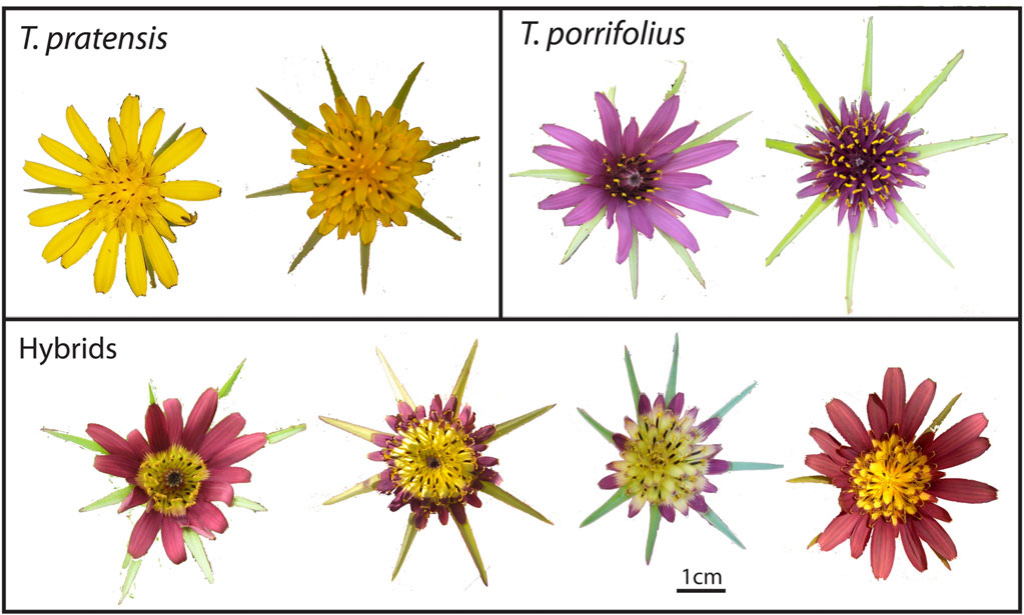
Typical inflorescences of *T. pratensis*, *T. porrifolius* and hybrids found in this study.

At least one parental species was found at each of these sites, and at two of the sites no hybrids were found. All putative hybrids had inflorescences with purple outer flowers and yellow inner flowers (Fig. 2), except for very rare cases with other intermediate morphology where purple and yellow coloration was irregularly mixed in each floret (e.g. Fig. 4). Collections of plants were made as follows: at Cannon Hill Common, 36 *T. porrifolius*, 15 *T. pratensis* and 16 hybrids; at Cooling Common, 13 *T. porrifolius*, 19 *T. pratensis* and 92 hybrids; at Hadleigh Downs, nine *T. porrifolius*, five *T. pratensis* and six hybrids; at Camley Road Nature Reserve, nine *T. porrifolius*; at Ranscombe Farm seven *T. pratensis*; at Decoys Lane, 75 *T. porrifolius*, two *T. pratensis* and two hybrids. A further 34 *T. porrifolius*, 42 *T. pratensis* and 12 hybrids were collected from among these six populations, whose location of origin was lost. Within the two parental species, both long and short ray floret morphs were present and sampled (Fig. 2).

### Flow cytometry

Fresh leaf samples were collected from plants identified by inflorescence morphology and stored in cool, moist conditions for up to 3 days. Flow cytometry was conducted on these fresh samples at the Jodrell Laboratory, Royal Botanic Gardens, Kew, to measure genome size (2C-value). *Tragopogon* leaf sections of ∼0.5 cm^2^ in size were co-chopped with leaves of the internal standard *Petroselinum crispum* ‘Curled Moss’ parsley [2C = 4.50 pg (Obermayer *et al*. 2002)], using a clean razor blade, in 1.5 mL of ice-cold ‘general plant isolation buffer’ (GPB, Loureiro *et al*. 2007) supplemented with 3 % polyvinylpyrrolidone (PVP-40). The homogenate was filtered through a 30-μm nylon mesh filter. The resulting nuclei suspension was stained with 0.5 μL of propidium iodide solution and supplemented with 0.8 μL of RNase to a final concentration of 50 μg mL^−1^. Samples were stored on ice for 15 min. The relative fluorescence of 1000 nuclei per sample was measured using a Partec GmbH PAII flow cytometer (Münster, Germany) fitted with a 100 W mercury arc lamp. The resulting histograms were analysed with the FlowMax software (v. 2.0, Partec GmbH) and the nuclear DNA contents estimated with the following formula: 2C-target = (target fluorescence peak/standard fluorescence peak) × 2C-standard.

### Statistical analyses on morphometric data

For each plant collected, we measured the following morphological traits: length of root, width of root at the top and at midpoint, length from the top of the root to the first branching node of the shoot and length from the top of the root to the tip of the main shoot. Counts of buds, inflorescences, secondary stems and tertiary stems were also recorded. Statistical analyses were performed on samples collected from Cannon Hill Common and Cooling Common as these both had sufficient numbers of *T. pratensis*, *T. porrifolius* and hybrids for site to be treated as a random effect. Each trait was investigated for normality with parametric and non-parametric tests of homogeneity of variances with Bartlett tests and Fligner–Killeen tests. The number of tertiary stems and the number of buds per plant were square-root transformed to meet assumptions of normality. Linear mixed effect (LME) models were fitted by residual maximum likelihood (REML) with the function lmer in the lme4 package (Bates *et al*. 2014). Each trait was fitted individually as a response variable, with plant group treated as a fixed effect and site treated as a random effect. An LME model with all nine traits simultaneously as response variables was also fitted to test for an overall significant difference among the three groups (i.e. *T. pratensis*, *T. porrifolius* and hybrids). Statistical analyses were implemented in R (R Development Core Team 2008).

After morphological data were collected, whole plants from each sampling site were pressed and dried using standard herbarium-sized blots and folders.

### Estimation of seed set

Where seed heads were available, we counted the number of plump and hollow (i.e. non-viable) achenes within each head. Plump achenes collected from some hybrids were tested for viability by placing them on damp filter paper in petri dishes, and germinants were planted into soil and grown to seedling stage.

### DNA sequence analyses

We extracted DNA from a subset of plants from our collections: 15 *T. porrifolius* plants, 14 *T. pratensis* plants and 6 hybrids. We also extracted DNA from a plant collected by Foucaud in France in 1889 and identified as *T. porrifolius* but may represent the syntype of *T.*×*mirabilis* by Rouy (1890) held by the Paris Herbarium (PO3290423); a *T. porrifolius* plant collected (R. Buggs) in Tuscany, Italy in 2011; a *T. pratensis* plant collected (K. Emelianova) in the French Alps in 2012; and both *T. porrifolius* (2677-5, collected in Pullman, WA, USA, by C. Cody 27 June 2005) and *T. pratensis* (2609-24, collected in Spangle, WA, USA in 15 July 1999 by D. & P. Soltis).

Extraction of DNA was done using a modified CTAB method (Doyle and Doyle 1987). Using PCR we amplified the internal transcribed spacer (ITS1 and ITS2) and external transcribed spacer (ETS) sequences (located between the 18S and 26S ribosomal RNA genes), alcohol dehydrogenase (*ADH*) and three plastid regions (Taberlet *et al*. 1991), and sequenced them using Sanger sequencing. The ETS, ITS and *ADH* sequences were placed into multi-species alignments of *Tragopogon* (Mavrodiev *et al*. 2005, 2007, 2015), and all sequences were compared on a site-by-site basis. The strategy of amplification of ITS, ETS, *ADH* and plastid loci followed that described in Mavrodiev *et al.* (2008*b*, 2012), including the listed primers. The *ADH* locus was amplified using the primer pair: *ADH*_F and *ADH*_R from Mavrodiev *et al*. (2012). Maximum likelihood (ML) analyses of the ITS and ETS datasets were conducted separately using PhyML v. 3.1 (Guindon *et al*. 2010) following the strategy described in Mavrodiev *et al*. (2014) using sequence data from Mavrodiev *et al.* (2008*b*, 2012).

All sequenced samples of *T. porrifolius* and *T. pratensis* were included in the phylogenetic analyses to check their identification. For hybrid plants, we compared all sequences on a site-by-site basis and presented all results in the format of Tables (see for example Mavrodiev *et al*. 2015 for a similar approach), because the presence of multiple polymorphic single-nucleotide polymorphisms (SNP) in the raw nuclear chromatograms may bias phylogenetic tree topologies (reviewed in Soltis *et al*. 2008).

### Karyotype of a putative backcross

The chromosomal composition of a putative hybrid individual from Cannon Hill Common with an unusual morphology and low genome size (see Results) was investigated. The plant was pulled from the soil and its roots were wrapped in wet sterile tissue paper and placed in a polythene bag. The plant was kept at room temperature in a well-lit lab and water added to the roots when the tissue paper began to dry. The terminal 2 cm of growing roots were harvested and pretreated in an aqueous solution of 2 mM 8-hydroxyquinoline for 16 h at 4 °C. Pretreated roots were then fixed in ice-cold 90 % acetic acid for 10 min and transferred to 70 % ethanol for −20 °C storage (Kato *et al*. 2011). Mitotic chromosome preparations and *in situ* hybridization were conducted with modifications to Kato *et al*. (2011) as described in Chester *et al*. (2012). Chromosome preparations were first subjected to fluorescence *in situ* hybridization (FISH) and then to genomic *in situ* hybridization (GISH). For FISH, probes comprised the following repetitive sequences: Cy5-labelled TPRMBO (Pires *et al*. 2004), Cy3-labelled TGP7 (Pires *et al*. 2004), Cy3- and fluorescein-labelled 18S rDNA and fluorescein-labelled TTR3 (Chester *et al*. 2013). For GISH, probes comprised Cy5-labelled total genomic DNA of *T. pratensis* (Colton, WA, USA; ID: 3939) and Cy3-labelled total genomic DNA of *T. porrifolius* (Pullman, WA, USA; ID: 3932). Image acquisition and processing, and karyotype construction were carried out as described previously (Chester *et al*. 2012). The parental origin of chromosomes was based on GISH signals in the centromeric and pericentromeric regions.

## Results

### Genome sizes

Flow cytometry was used to estimate genome sizes (2C) of 41 *T. pratensis*, 110 *T. porrifolius* and 94 putative hybrid plants. The genome sizes of *T. pratensis* plants ranged from 4.97 to 5.22 pg (mean = 5.14 pg; SD = 0.042), those of *T. porrifolius* ranged from 6.16 to 6.43 pg (mean = 6.28 pg; SD = 0.047) and those of the hybrids ranged from 5.38 to 5.83 pg (mean = 5.72 pg; SD = 0.064). The means of the three groups differed significantly (*F*_2,242_ = 7326, *P* < 0.00001). If the hybrids were *F*_1_ hybrids between these two species, we would expect them to have genome sizes between 5.57 and 5.83 pg, based on the maximum and minimum additive genome sizes of the parents. The lower than expected minimum range of the hybrids' distribution, and their higher standard deviation, was caused by two plants with smaller genome sizes than the others, being 5.38 and 5.54 pg. When these were excluded, the 2C-values of hybrids ranged from 5.61 to 5.83 pg (mean = 5.72 pg; SD = 0.050), as expected. One of the hybrids with a low genome size (5.54 pg), plant 1000 from Cannon Hill Common, was investigated further using cytogenetic methods (see below). All plants tested had genome sizes within the known diploid range of *Tragopogon*, so none of the plants sampled was polyploid.

### Morphometric analysis

**Figure 3.**
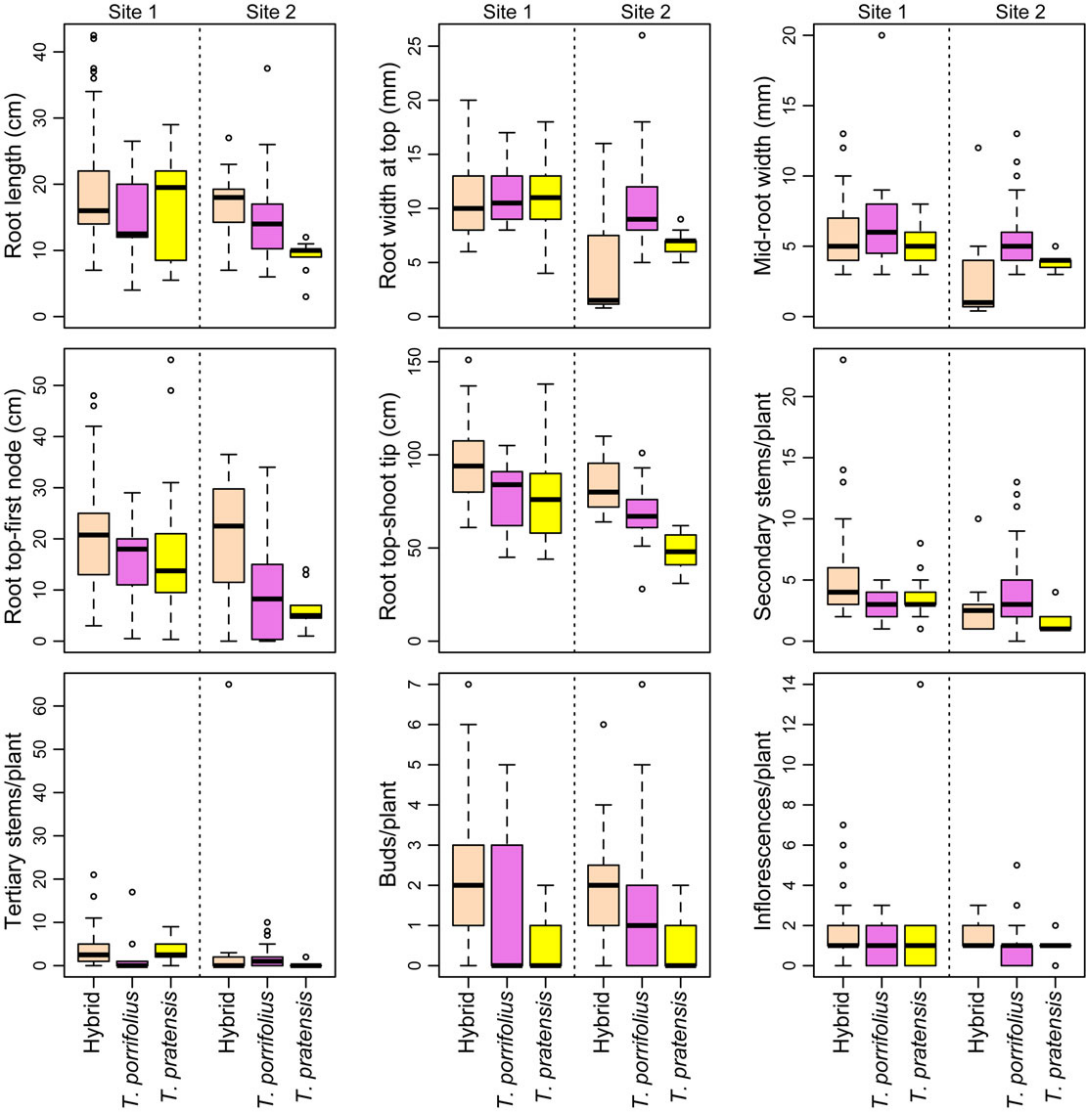
Box-and-whisker plots of morphological traits of *T. porrifolius*, *T. pratensis* and their hybrid collected from Cooling Common (Site 1) and Cannon Hill Common (Site 2). Outliers are shown as circles.

Linear mixed effect models comparing *T. porrifolius* and *T. pratensis* with the hybrid taxon, with site as a random effect, were carried out on 9 *T. pratensis* plants, 42 *T. porrifolius* plants and 16 hybrids from Cannon Hill Common and 18 *T. pratensis* plants, 13 *T. porrifolius* plants and 86 hybrids from Cooling Common. After Bonferroni correction, these showed the hybrids to differ from *T. porrifolius* in having smaller root width at top and middle, and in being greater in length from the top of the root to the first stem node (Table 1 and Fig. 3). The hybrids were greater than both parental species in length from the top of the root to the tip of the shoot, and in the number of buds per plant (Table 1 and Fig. 3). An LME examining the response of all nine traits shown in Fig. 3, to species, with site as a random effect, showed a significant difference among the three plant groups, with the hybrid significantly different from both parents (*T. porrifolius*: *P* = 0.0172, *T. pratensis*: *P* = 0.0463) (Table 1).

**Table 1.** Linear mixed effect models showing *T. porrifolius* and *T. pratensis* compared with the hybrid taxon. Number of tertiary stems and number of buds per plant were square-root transformed. d.f., degrees of freedom.

Response variable	Species	Value	Standard error	d.f.	*t*-value	*P*-value
Root length	(Intercept)	17.691	1.375	171	12.862	<0.0001
	*T. porrifolius*	−2.362	1.448	171	−1.632	0.1046
	*T. pratensis*	−3.886	1.600	171	−2.429	0.0162
Root width at top	(Intercept)	8.432	1.906	177	4.424	<0.0001
	*T. porrifolius*	3.004	0.765	177	3.927	0.0001
	*T. pratensis*	0.479	0.827	177	0.580	0.5627
Mid-root width	(Intercept)	4.448	0.924	171	4.813	<0.0001
	*T. porrifolius*	2.185	0.514	171	4.249	<0.0001
	*T. pratensis*	−0.146	0.550	171	−0.265	0.7911
Root base-first node	(Intercept)	18.754	2.299	180	8.157	<0.0001
	*T. porrifolius*	−6.897	1.933	180	−3.568	0.0005
	*T. pratensis*	−5.535	2.156	180	−2.567	0.0111
Root base-shoot tip	(Intercept)	88.747	7.835	180	11.327	<0.0001
	*T. porrifolius*	−14.314	3.553	180	−4.028	<0.0001
	*T. pratensis*	−23.504	3.889	180	−6.043	<0.0001
Secondary stems	(Intercept)	4.467	0.571	180	7.821	<0.0001
	*T. porrifolius*	−0.362	0.561	180	−0.645	0.5195
	*T. pratensis*	−1.454	0.633	180	−2.295	0.0229
sqrt(Tertiary stems)	(Intercept)	1.365	0.257	180	5.315	<0.0001
	*T. porrifolius*	−0.332	0.225	180	−1.477	0.1416
	*T. pratensis*	−0.261	0.251	180	−1.036	0.3015
sqrt(Buds/plant)	(Intercept)	1.332	0.071	180	18.692	<0.0001
	*T. porrifolius*	−0.625	0.120	180	−5.194	<0.0001
	*T. pratensis*	−0.870	0.156	180	−5.583	<0.0001
Inflorescences/plant	(Intercept)	1.696	0.155	180	10.910	<0.0001
	*T. porrifolius*	−0.660	0.263	180	−2.512	0.0129
	*T. pratensis*	−0.141	0.340	180	−0.414	0.6797
All nine morphometric	*(Intercept)*	68889155	15339444	172	4.491	<0.0001
variables	*T. porrifolius*	−62878560	26130961	172	−2.406	0.0172
	*T. pratensis*	−65969696	32872942	172	−2.007	0.0463

### Achene production

We counted the number of achenes in one complete head from each of 69 *T. porrifolius* plants, 17 *T. pratensis* plants and 65 hybrids; the mean numbers of achenes per head were 73.5 (SD = 25.7), 47.1 (SD = 19.4) and 62.2 (SD = 17.1), respectively. For a further 25 *T. porrifolius*, 45 *T. pratensis* and 21 hybrid plants, we could only count achenes from heads that were incomplete because of loss of achenes due to dispersal or disturbance. In all heads, we calculated the proportions of achenes that were plump versus those that were hollow (i.e. aborted). The mean percentage of aborted achenes was found to be: 24.1 % (*n* = 94, SD = 37.4) in *T. porrifolius* plants, 7.9 % (*n* = 62, SD = 16.3) in *T. pratensis* plants and 90.4 % (*n* = 86, SD = 11.3) in hybrids; these results were significantly different (Kruskal–Wallis test: *K* = 3, *H* = 111.1, *P* < 0.0001). The notably high standard deviation for the percentage of aborted seeds in *T. porrifolius* was due to a subset of 15 *T. porrifolius* plants in which all seeds were aborted. It should be noted that poor reproductive success in the hybrid due to seed abortion was partly mitigated by higher production of inflorescence buds (Table 1).

Some of the achenes produced by plants whose hybrid status was suggested by their genome size and morphology were successfully germinated and grown to seedling stage. Hybrid plant 312 from Cannon Hill Common produced 28 achenes, of which 7 were plump, and 3 germinated, producing seedlings. Hybrid plant 403 from Cooling Common (which was also shown to be hybrid by DNA sequence analysis; see below) had 80 achenes, of which 6 were plump, and only 1 produced a seedling. Hybrid plant 404 from Cooling Common had 94 achenes, of which 13 were plump and 7 produced seedlings.

### DNA sequence analyses

The five hybrids for which we obtained *ADH* sequences all showed the presence of double peaks at sites that differentiate the parents, corresponding to the bases present in both *T. porrifolius* and *T. pratensis* plants (Table 2): this is consistent with an *F*_1_ hybrid status of these plants. For their plastid loci, the hybrids only had haplotypes found in *T. pratensis* (Table 2), which indicates that *T. pratensis* is the maternal parent of all five of the hybrids analysed. Plant 3447, which had *T. porrifolius* morphology, showed two unusual base variants in its *ADH* sequence that were not found in any other plants analysed.

**Table 2.** Molecular analyses showing plant collection details, genotypes at plastid (maternally inherited) and ADH (nuclear) variable sites and whether the samples were included in the ITS and ETS analyses.

Morphology	Location	DNA number	Collection number	Nucleotide calls at variable plastid sites			Nucleotide calls at variable ADH sites								ETS	ITS
				205–211	1184–1193	1221	93	261	406	441	631	639	667	685		
*Porrifolius*	Camley Road	3481	CRNR13	–	–	T										
*Porrifolius*	Camley Road	3486	CRNR14	–	–	T										
*Porrifolius*	Camley Road	3480	CRNR3	–	–	T										
*Porrifolius*	Cannon Hill Common	3449	311				A	T	T	A	C	T	C	A	Y	
*Porrifolius*	Cannon Hill Common	3447	329				A	C	C	A	C	T	C	A	Y	Y
*Porrifolius*	Cannon Hill Common	3477	307	–	–	T										
*Porrifolius*	Cannon Hill Common	3478	310	–	–	T										
*Porrifolius*	Cannon Hill Common	3440	322	–	–	T									Y	Y
*Porrifolius*	Cannon Hill Common	3441	809	–	–	T									Y	
*Porrifolius*	Cannon Hill Common	3442	817												Y	
*Porrifolius*	Cannon Hill Common	3474/77	321	–	–	T										
*Porrifolius*	Cooling Common	3450	555				A	T	T	A	C	T	C	A	Y	Y
*Porrifolius*	Cooling Common	3448	621				A	T	T	A	C	T	C	A	Y	
*Porrifolius*	Cooling Common	3475	556	–	–	T										
*Porrifolius*	Decoys Lane	3444	643				A	T	T	A	C	T	C	A	Y	
*Porrifolius*	Decoys Lane	3443	651												Y	
*Porrifolius*	Hadleigh Downs	3445	427				A	T	T	A	C	T	C	A	Y	
*Porrifolius*	Tuscany, Italy	3483	Tuscany	–	–	T									Y	
*Porrifolius*	France, Foucaud, 1889	3790/09	PO3290423												Y	
*Pratensis*	Cannon Hill Common	3454	339				G	T	T	G	A	G	T	A	Y	
*Pratensis*	Cannon Hill Common	3485	317	ATTTTTG	TTATACAAAT	T										
*Pratensis*	Cannon Hill Common	3451	337	ATTTTTG	TTATACAAAT	G									Y	
*Pratensis*	Cannon Hill Common	3470	302	ATTTTTG	TTATACAAAT	T										
*Pratensis*	Cannon Hill Common	3473	304	ATTTTTG	TTATACAAAT	T										
*Pratensis*	Cannon Hill Common	3469	305	ATTTTTG	TTATACAAAT	G										
*Pratensis*	Cooling Common	3453	551				G	T	T	G	A	G	T	A	Y	Y
*Pratensis*	Cooling Common	3452	552				G	T	T	G	A	G	T	A	Y	
*Pratensis*	Cooling Common	3455	553				G	T	T	G	A	G	T	A	Y	
*Pratensis*	Cooling Common	3462	554	ATTTTTG	TTATACAAAT	G									Y	
*Pratensis*	Cooling Common	3467/82	550	ATTTTTG	TTATACAAAT	G									Y	
*Pratensis*	Hadleigh Downs	3468	443	ATTTTTG	TTATACAAAT	T										
*Pratensis*	Ranscombe Farm	3460	600	ATTTTTG	TTATACAAAT	G										
*Pratensis*	Ranscombe Farm	3463	601	ATTTTTG	TTATACAAAT	G										
*Pratensis*	French Alps	3464	KE001	ATTTTTG	–	T									Y	
Hybrid	Cooling Common	3446	63	ATTTTTG	TTATACAAAT	G	A/G	T	T	A/G	M	T/G	C/T	A	Y	Y
Hybrid	Cooling Common	3458	65	ATTTTTG	TTATACAAAT	G	A/G	T	T	A/G	M	T/G	C/T	A	Y	Y
Hybrid	Cooling Common	3456	403	ATTTTTG	TTATACAAAT	G	A/G	T	T	A/G	M	T/G	C/T	A	Y	
Hybrid	Cooling Common	3457	409	ATTTTTG	TTATACAAAT	G	A/G	T	T	A/G	M	T/G	C/T	A	Y	
Hybrid	Cooling Common	3459	408	ATTTTTG	TTATACAAAT	G	A/G	T	T	A/G	M	T/G	C/T	A	Y	
Hybrid	Cannon Hill Common	3484	RB186												Y	

Phylogenetic analyses of ITS and ETS sequence data showed that in almost full agreement with previous results (e.g. Mavrodiev *et al*. 2005, 2008*b*) non-hybrids sequenced from southeast England appeared to be *T. pratensis* or *T. porrifolius* subspecies *porrifolius* sensu Flora Europaea (Richardson 1976): i.e. they likely come from the most widespread lineages of each species (see Introduction). Some samples of *T. porrifolius* possessed from one to few polymorphic SNPs in their ITS (Table 3) and/or ETS (Table 4) sequences, perhaps due to the incomplete homogenization of the repeats between two rDNA loci; this may be evidence for past hybridization. The 1889 collection from France (PO3290423), named as *T. porrifolius* by Foucaud, and possibly as *T.*×*mirabilis* by Rouy (1890) showed numerous double peaks at ETS sites (Table 4) that may suggest a hybrid origin, but as it contained six SNPs in the ETS region that were not found in any of the other plants we sampled, it is unlikely to be a hybrid between *T. pratensis* and *T. porrifolius* unless considerable nucleotide divergence has occurred in space and/or time within the species. In the ETS phylogenetic reconstruction, it was found in the Brevirostres clade **[see Supporting Information—Fig. S2]**.

**Table 3.** Summary of ITS base calls at variable sites.

	26	34	58	88	90	101	107	411	425	439	497	519
*T. porrifolius* Pullman (USA), 3440, 3442, 3443, 3444, 3445, 3448, 3449 (UK)	A/T	T	A	A	C	C	T	G	T/G	T	C	C
*T. porrifolius* 3248 (UK)	A/T	T	A	A	C	C	T	G	T	T	C	C
*T. porrifolius* 3450 (UK)	A	T	A	A	C	C	T	G	T	T	C	C
*T. porrifolius* 3447, 3441 (UK)	A/T	T	A/G	A/G	C/A	C	C/T	A/G	T	C/T	C/T	C/T
Hybrids 3446, 3456, 3457, 3459 (UK)	A/T	T	A/G	A/G	C/A	C	C/T	A/G	T/G	C/T	C/T	C/T
Hybrids 3458 (UK)	A/T	T/G	A/G	A/G	C/A	C	C/T	A/G	T/G	C/T	C/T	C/T
*T. pratensis* Spangle (USA)	A	T	G	G	A	T	C	A	T	C	T	T
*T. pratensis* 3455, 3451, 3452, 3453, 3454, 3245	A	T	G	G	A	C	C	A	T	C	T	T

**Table 4.** Summary of ETS base calls at variable sites.

	18	19	44	55	71	147	150	195	200	202	203	212	219	223	278	309	361	362	411	417	428	509
*T. porrifolius* 3483 (Italy)	C	T	C	G	G	T	T	C	A	A	G	G	G	G	A	G	T	G	T	C	A	T
*T. porrifolius* Pullman (USA) and 3440, 3442, 3443, 3444, 3445, 3448, 3449, 3450 (UK)	C	T	C	G	G	T	T	C	A	A	A/G	T/G	G	G	A	G	T	G	T	C	A	T
*T. porrifolius* 3447 and 3441 (UK)	C/T	G/C	C/T	G	A/G	C/T	C/T	C	A/T	A/T	G	T/G	T/G	G	C/A	A/G	T	A/G	T	C/T	A	T
Foucaud collection 3709 PO3290423 (France, 1889)	C	T	C	G	G	C/T	T	C/A	A	A/T	G	T/G	G	A/G	A	G	C	G	C	C	T	A/T
Hybrid 3484 (Cannon Hill Common, UK)	C	T	C/T	T/G	A/G	C/T	C/T	C	A/T	A/T	G	T/G	T/G	G	C/A	A/G	T	A/G	T	C	A	T
Hybrid 3446, 3456, 3457, 3458, 3459 (Cooling Common, UK)	C	T	C/T	T/G	A/G	C/T	C/T	C	A/T	A/T	A/G	T/G	T/G	G	C/A	A/G	T	A/G	T	C	A	T
*T. pratensis* Spangle (USA), 3464 (France), 3460, 3461 (UK)	C	T	T	G	A	C	C	C	T	T	G	G	T	G	C	A	T	A	T	C	A	T
*T. pratensis* 3462, 3463, 3465, 3466, 3467, 3451, 3452, 3453, 3454, 3455, 3467, 3485 (UK)	C	T	T	T	A	C	C	C	T	T	G	G	T	G	C	A	T	A	T	C	A	T

### Cytogenetic analysis of a putative backcross

**Figure 4.**
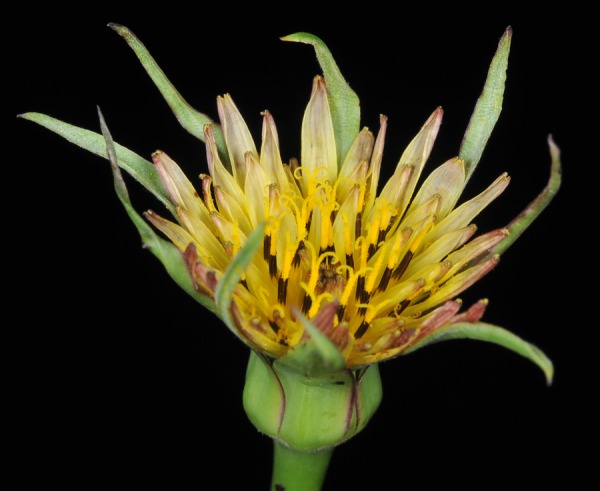
Inflorescence of plant 1000 from Cannon Hill Common, showing a morphology intermediate between that of hybrids and *T. pratensis*; the genome size of this plant showed a similar intermediacy.

**Figure 5.**
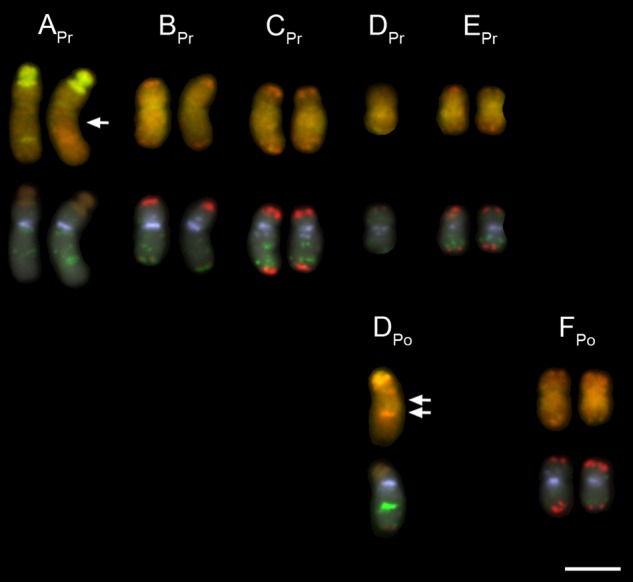
Karyotype of plant 1000. Each chromosome is shown twice, with signals resulting from either GISH (above) or FISH (below). Genomic *in situ* hybridization produced considerable cross-hybridization between genomes; chromatin of *T. pratensis* chromosomes appeared green/brown and chromatin of *T. porrifolius* chromosomes appeared red/orange. Fluorescence *in situ* hybridization allowed the chromosomes to be assigned to each homeologous group (A–F). Together, FISH and GISH revealed a skewed chromosome composition, with a bias towards *T. pratensis*. Chromosomes A_Pr_ (A from *T. pratensis*) and D_Po_ (D from *T. porrifolius*) showed intergenomic translocations (breakpoint positions are indicated by arrows). Fluorescence *in situ* hybridization probes were pseudo-coloured as follows: TGP7 (red), 18S rDNA (brown), TPRMBO (light blue), TTR3 (green). Chromosomes were counterstained with DAPI (grey). Scale bar: 5 μm.

The putative hybrid plant (1000) from Cannon Hill Common had inflorescence morphology (Fig. 4) and 2C-value (5.54 pg) intermediate between that of a typical hybrid and *T. pratensis*. It was investigated using *in situ* hybridization to resolve its genomic composition (Fig. 5). This revealed the chromosome number to be 2*n* = 12, but the complement did not match that expected of an *F*_1_ hybrid, confirming the likely backcross status of the plant. Only one of the six chromosomes (belonging to group D sensu Chester *et al*. 2013) showed the expected 1 : 1 (*T. pratensis:T. porrifolius*) parental ratio. The other chromosomes were found in either a 2 : 0 (Group A, B, C and E) or 0 : 2 ratio (Group F), resulting in a bias in chromosome composition towards *T. pratensis* chromosomes. Several non-reciprocal intergenomic translocations were also observed (see arrows, Fig. 5), with one breakpoint on chromosome A originating from *T. pratensis* (A_Pr_) and two breakpoints on the single D chromosome originating from *T. porrifolius* (D_Po_) chromosome. Although GISH differentiation was poor due to the high amount of cross-hybridization between genomes, chromosome identification was supported by the FISH signals [i.e. by the presence of diagnostic TPRMBO, TTR3 and 18S rDNA signals that differ in their distribution between progenitor chromosomes (Chester *et al*. 2013)]. For both the A and D chromosome translocations, FISH signals were also consistent with the translocations involving homeologous exchanges.

## Discussion

We found natural hybrids between *T. pratensis* and *T. porrifolius* in four populations in southeast England*.* At least one parental species was also found in each of these populations, and two sites that had previously been reported as containing hybrids were found to contain only plants with the morphology of the parental species. The hybrid plants had a morphology and fertility that fitted with the descriptions published by Linnaeus for this cross in 1760, and by numerous field and experimental botanists since (see Introduction). The *F*_1_ hybrid status of these plants was strongly supported by the flow cytometry data and confirmed by Sanger sequencing of genomic DNA regions. All *F*_1_ hybrids from which we sequenced plastid genes showed *T. pratensis* to be their maternal parent. None of the plants were polyploid. Together, these results suggest that the hybrids we found between *T. pratensis* and *T. porrifolius* are mainly ephemeral first-generation hybrids that have not speciated either at the homoploid or allopolyploid levels.

We found some evidence that backcrossing of the *F*_1_ hybrids may be occurring. Some hybrid plants produced low numbers of viable seeds, which germinated to produce seedlings. We investigated one plant with a genome size intermediate between those of *F*_1_ hybrids and *T. pratensis*, and found it to have a genome composition that could only have arisen via further rounds of meiosis since an *F*_1_. Unexpected DNA base variation in some plants that we initially identified as *T. porrifolius* may also be a consequence of hybridization followed by backcrossing. Thus, the hypothesis that gene flow may occur between *T. pratensis* and *T. porrifolius* via their hybrids merits further investigation.

Due to the low sample sizes and restricted sampling area of this study, our conclusions that speciation has not occurred but that some backcrossing is possible are obviously preliminary and restricted to those populations we sampled. However, we can find no records of natural homoploid hybrid species or allopolyploids between *T. pratensis* and *T. porrifolius*, despite frequent reports of hybridization both in their native European range (Gosselman 1864; Focke 1881; Thedenius 1885; Rouy 1890; Clausen 1966; Burrow and Burrow 1978; Stace 2010) and their introduced range in North America (Halsted 1890; Sherff 1911; Farwell 1930; Ownbey 1950; Clausen 1966). While broader surveys of larger numbers of individuals in both Europe and North America will be needed to fully confirm these conclusions, the fact that no new homoploid hybrid or allopolyploid species have been reported for this cross, despite the extensive botanical literature for Europe and North America suggests that our conclusions for southeast England may be true globally. Our findings are also remarkably similar to those of a succession of botanists who have identified *T. pratensis*, *T. porrifolius* and their hybrids in the field, or experimented on them, over the last 250 years. In 1966, Jens Clausen was struck by the stability of characters in *T. pratensis*, *T. porrifolius* and their hybrid over 200 years, concluding that there is ‘a high degree of permanence of the basic genetic structure of species’ (Clausen 1966, p. 157).

Assuming that these conclusions prove to be correct, hybrids between *T. pratensis* and *T. porrifolius* may be a useful study system to address the question of why three other possible outcomes have not evolved: (i) allopolyploid speciation; (ii) homoploid hybrid speciation; and (iii) divergence between *T. pratensis* and *T. porrifolius* to prevent hybridization. We outline these research questions below.

### Why have allopolyploids not formed?

The lack of allopolyploids between *T. pratensis* and *T. porrifolius* is perhaps notable given that natural hybridization of both *T. pratensis* and *T. porrifolius* with *T. dubius* has yielded allopolyploid species on several independent occasions in the last 100 years (Ownbey 1950; Soltis *et al*. 2004; Symonds *et al*. 2010), and allopolyploids between *T. pratensis* and *T. porrifolius* have been produced by artificial hybridization and colchicine treatment in the glasshouse from American diploid plants (Tate *et al*. 2009).

One factor may be that *T. pratensis* and *T. porrifolius* are more closely related to one another than either of these species is to *T. dubius* **[see Supporting Information—Figs S1 and S2]**. As reviewed in Buggs *et al*. (2011*a*), it has long been suggested that hybridization between divergent parental species may promote polyploidization. The relationship between parental divergence, hybridization and polyploidy has been discussed in the last decade (Chapman and Burke 2007; Buggs *et al*. 2008, 2009, 2011*a*; Paun *et al*. 2009, 2011), mainly relying on statistical comparisons of parental divergence of homoploid hybrids and allopolyploids in several plant genera. The findings of this paper do not directly add to this discussion, as the discussion's various statistical analyses have already included the different outcomes of crossing in the *Tragopogon* triangle investigated here. However, the *Tragopogon* triangle might provide a useful study system to investigate mechanical hypotheses for how divergence might affect the outcomes of hybridization: for example, the possibility that patterns of divergence at particular loci or in particular chromosomal arrangements in *T. pratensis*, *T. porrifolius* and *T. dubius* may be affecting the outcomes of hybridization.

Another possibility that might be investigated is that *T. dubius* carries alleles that cause it to have a greater proclivity for allopolyploidization than *T. pratensis* and *T. porrifolius*. The existence of genetic variants promoting polyploidization was suggested by Grant (1981) and is shown by the success of selective breeding for rates of 2*n* gamete formation in *Medicago* and *Trifolium* (Grant 1981; Ramsey and Schemske 1998).

The influence of historical or biogeographic factors on hybridization in this system also warrants further investigation as they are likely to have a major role. *Tragopogon pratensis* and *T. porrifolius* are rarer in the Palouse area of Washington and Idaho in the USA than *T. dubius*, so there may have been fewer opportunities for hybridization between *T. porrifolius* and *T. pratensis.* The fact that *Tragopogon* allopolyploids have formed in Washington and Idaho but not in Europe may be because occasional environmental shocks such as extreme frosts during flowering (Hagerup 1932; Ramsey and Schemske 1998) have occurred more in Washington and Idaho and induced chromosome doubling. It could also be the case that ecological niches suitable for *T. pratensis*×*T. porrifolius* allopolyploids have not been available.

### Why has homoploid hybrid speciation not occurred?

This question is perhaps easier to answer because although new homoploid hybrid species may evolve, the conditions required for their establishment are more stringent than the conditions for allopolyploid species establishment (Buerkle *et al*. 2000), as homoploid hybrids do not benefit from the immediate escape from parental gene flow that polyploids usually enjoy (Stebbins 1950). Models suggest that homoploid hybrid species can only evolve if they have sufficient ecological and spatial isolation from their parental species (Buerkle *et al*. 2000), due to ecological selection (Gross and Rieseberg 2005), as seems to be the case for homoploid hybrid species of *Helianthus* (Rieseberg *et al*. 2003) and *Senecio* (Abbott *et al*. 2010). The hybrids found in this study were all growing in similar habitats to the parental species and in close proximity to them; they differed in some morphological traits, being on average transgressive in height and number of buds produced, and were significantly different in an LME model that took all of our morphological measurements into account. Homoploid hybrids are most likely to have evolutionary independence from their parents if they have chromosomal rearrangements that cause them to be reproductively isolated from their parental species (Buerkle *et al*. 2000; Yakimowski and Rieseberg 2014). The backcross hybrid that we karyotyped shows chromosomal variations that may provide incompatibilities with the parents, but isolation from parents by itself is not sufficient to cause speciation (Buerkle *et al*. 2000; Yakimowski and Rieseberg 2014).

### Why have *T. pratensis* and *T. porrifolius* not diverged further?

If two species can hybridize to form low-fitness hybrids, there should be selective pressures causing reinforcement of pre-zygotic isolation mechanisms between the hybridizing species (Dobzhansky 1940). Although this hypothesis has been criticized (e.g. Howard 1993; Marshall *et al*. 2002), other evidence supports it (e.g. Hopkins and Rausher 2011; Andrew and Rieseberg 2013). In the present study, we find no evidence for ongoing reinforcement as *T. pratensis* and *T. porrifolius* appear to have been hybridizing and producing low-fertility hybrids over at least the last 250 years. This may be because 250 years is too short a timespan for further divergence to have evolved, being only 125 generations of these biennial species (c.f. Buerkle and Rieseberg 2008). It may be that the rate of hybrid production is too low to be of significant reproductive cost to either species, or that sites of hybrid production have been ephemeral so that selection has not acted consistently on particular populations for extended periods. Alternatively, it is well known that gene flow can prevent the divergence of species (Slatkin 1987), and it could be that hybridization and concomitant gene flow between *T. porrifolius* and *T. pratensis*, though very low, are sufficiently high to hinder increased divergence between the two species. If this were the case, it would appear that levels of gene flow are low enough not to cause merging of the species. It may also be that natural selection is maintaining the two species in the face of gene flow (Nosil 2008; Abbott *et al*. 2013). Thus, it could be worth investigating whether the two species appear stable due to a dynamic process of gene flow that, in balance with natural selection on the two parental morphs, is holding the system in a dynamic equilibrium.

## Conclusions

*Tragopogon* has been extensively developed as a model system to study the genomics and transcriptomics of allopolyploid speciation, where rapid change has been shown to occur both in the formation of the allopolyploids and in their subsequent generations (Tate *et al*. 2006; Buggs *et al*. 2011*b*, 2012; Chester *et al*. 2012; Soltis *et al*. 2012; Lipman *et al*. 2013). In contrast, although hybrids between *T. pratensis* and *T. porrifolius* have been studied scientifically for a longer period than any other plant hybrid, over this 250-year period of experimentation and observation there appears to have been little outward change in the dynamics of this interaction and its morphological consequences. In this paper we speculate as to why this is so, but thorough understanding of the interaction, and particularly of the dynamics of gene flow, which may be critical to the apparent stability of the parental species, will only come through genome-wide analyses of variation in natural populations. The present study lays the foundations for such future research. Understanding why hybrids do not speciate, despite repeated opportunities, would enhance our understanding of both the evolutionary process and risk assessments of invasive species. The apparent stasis of the diploid species and their hybrids in the present study underlines the importance of polyploidy in the promotion of rapid evolution in this genus.

## Accession Numbers

Herbarium samples of representative material are deposited with the British Museum Herbarium with accession numbers BM001139296–BM001139307 **[see Supporting Information—Table S1]**.

The DNA sequences have been deposited in GenBank with accession numbers as follows: KT167073–KT167093 (ITS sequences), KT167094–KT167124 (ETS sequences), KT167125–KT167149 (plastid sequences) and KT167150–KT167161 (*ADH* sequences).

## Sources of Funding

This work was principally supported by SYNTAX grant 2010/11 number 13, administered by the Linnean Society of London and funded by NERC and BBSRC, and by NERC Fellowship NE/G01504X/1. Molecular and cytogenetic work was supported by NSF grants DEB-0922003 and DEB-1146065.

## Contributions by the Authors

R.J.A.B., E.V.M., A.R.L., I.J.L., M.C., D.E.S. and P.S.S. formed the research questions. A.M., K.E., M.C., A.A.H., J.P., K.S.A., M.S.G., E.V.M. and R.J.A.B. implemented the project in the field and laboratory. All authors contributed to the development, analysis of data and manuscript drafting.

## Conflict of Interest Statement

None declared.

## Supporting Information

The following additional information is available in the online version of this article —

**Figure S1.** Maximum likelihood ITS tree of the genus *Tragopogon* showing hybrid samples forming a clade with *T. porrifolius.* Samples from the present study have a four-digit ID number in their label.

**Figure S2.** Maximum likelihood ETS tree of the genus *Tragopogon* showing hybrid samples forming a clade with *T. pratensis*, and unexpected placement of the accession labelled *T. porrifolius* from the Paris herbarium. Samples from the present study have a four-digit ID number in their label.

**Table S1.** List of herbarium specimens deposited at the British Museum Herbarium with accession numbers.

Additional Information
