# Supplementary material for: 250 years of hybridization between two biennial herb species without speciation
Source: AoB Plants. 2015 Jul 17;7:plv081. doi: 10.1093/aobpla/plv081 (PMC4571729; doi:10.1093/aobpla/plv081)
Supplement: Additional Information [file supp_7_plv081_index.html]

250 years of hybridization between two biennial herb species without speciation — Additional Information 

# 250 years of hybridization between two biennial herb species without speciation

## Additional Information

Additional Information

- Supplementary Figures - pdf file
- Supplementary Table 1 - docx file
