# Supplementary material for: 250 years of hybridization between two biennial herb species without speciation
Source: AoB Plants. 2015 Jul 17;7:plv081. doi: 10.1093/aobpla/plv081 (PMC4571729; doi:10.1093/aobpla/plv081)
Supplement: Additional Information [file supp_plv081_plv081supp_figs.pdf]

SUPPORTING INFORMATION

The following Supporting Information is available in the online version of this article.

File 1: Figure S1. Maximum Likelihood ITS tree of the genus *Tragopogon* showing hybrid samples forming a clade with *T. porrifolius.* Samples from the present study have a four-digit ID number in their label.

File 2: Figure S2. Maximum Likelihood ETS tree of the genus *Tragopogon* showing hybrid samples forming a clade with *T. pratensis*, and unexpected placement of the accession labelled *T. porrifolius* from the Paris herbarium. Samples from the present study have a four-digit ID number in their label.

File 3: Table S1. List of herbarium specimens deposited at the British Museum Herbarium with accession numbers.

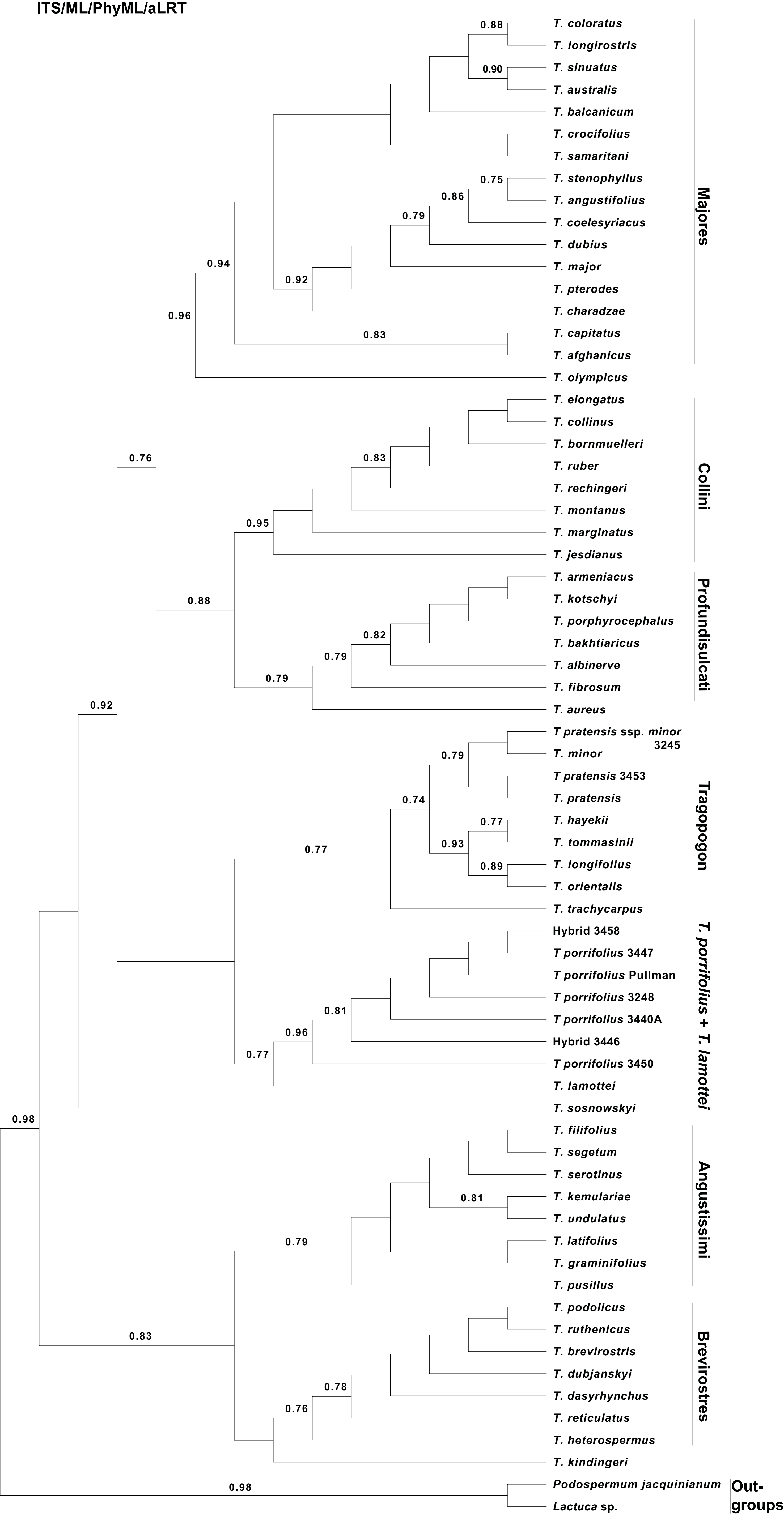
ITS/ML/PhyML/aLRT
T. coloratus
T. longirostris
T. sinuatus
T. australis
T. balcanicum
T. crocifolius
T. samaritani
T. stenophyllus
T. angustifolius
T. coelesyriacus
T. dubius
T. major
T. pterodes
T. charadzae
T. capitatus
T. afghanicus
T. olympicus
T. elongatus
T. collinus
T. bornmuelleri
T. ruber
T. rechingeri
T. montanus
T. marginatus
T. jesdianus
T. armeniacus
T. kotschyi
T. porphyrocephalus
T. bakhtiaricus
T. albinerve
T. fibrosum
T. aureus
T pratensis ssp. minor 3245
T. minor
T pratensis 3453
T. pratensis
T. hayekii
T. tommasinii
T. longifolius
T. orientalis
T. trachycarpus
Hybrid 3458
T porrifolius 3447
T porrifolius Pullman
T porrifolius 3248
T porrifolius 3440A
Hybrid 3446
T porrifolius 3450
T. lamottei
T. sosnowskyi
T. filifolius
T. segetum
T. serotinus
T. kemulariae
T. undulatus
T. latifolius
T. graminifolius
T. pusillus
T. podolicus
T. ruthenicus
T. brevirostris
T. dubjanskyi
T. dasyrhynchus
T. reticulatus
T. heterospermus
T. kindingeri
Podospermum jacquinianum
Lactuca sp.
Majores
Collini
Profundisulcati
Tragopogon
T. porrifolius + T. lamottei
Angustissimi
Brevirostres
Out-groups
0.88
0.90
0.75
0.86
0.79
0.94
0.92
0.96
0.83
0.76
0.83
0.95
0.88
0.82
0.79
0.79
0.92
0.79
0.74
0.77
0.93
0.77
0.89
0.81
0.96
0.77
0.98
0.81
0.79
0.83
0.78
0.76
0.98

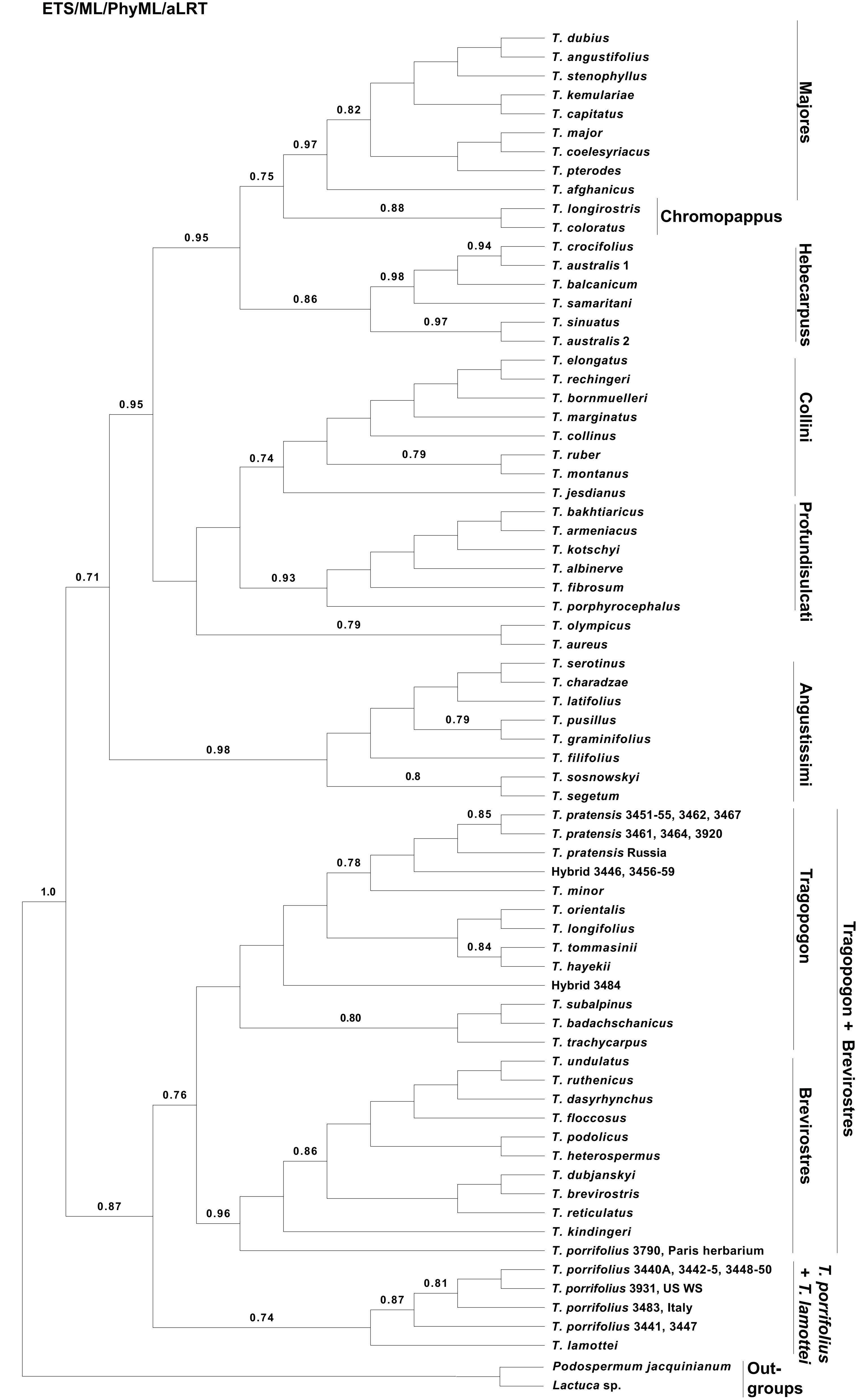

ETS/ML/PhyML/aLRT
T. dubius
T. angustifolius
T. stenophyllus
T. kemulariae
T. capitatus
T. major
T. coelesyriacus
T. pterodes
T. afghanicus
T. longirostris
T. coloratus
T. crocifolius
T. australis 1
T. balcanicum
T. samaritani
T. sinuatus
T. australis 2
T. elongatus
T. rechingeri
T. bornmuelleri
T. marginatus
T. collinus
T. ruber
T. montanus
T. jesdianus
T. bakhtiaricus
T. armeniacus
T. kotschyi
T. albinerve
T. fibrosum
T. porphyrocephalus
T. olympicus
T. aureus
T. serotinus
T. charadzae
T. latifolius
T. pusillus
T. graminifolius
T. filifolius
T. sosnowskyi
T. segetum
T. pratensis 3451-55, 3462, 3467
T. pratensis 3461, 3464, 3920
T. pratensis Russia
Hybrid 3446, 3456-59
T. minor
T. orientalis
T. longifolius
T. tommasinii
T. hayekii
Hybrid 3484
T. subalpinus
T. badachschanicus
T. trachycarpus
T. undulatus
T. ruthenicus
T. dasyrhynchus
T. floccosus
T. podolicus
T. heterospermus
T. dubjanskyi
T. brevirostris
T. reticulatus
T. kindingeri
T. porrifolius 3790, Paris herbarium
T. porrifolius 3440A, 3442-5, 3448-50
T. porrifolius 3931, US WS
T. porrifolius 3483, Italy
T. porrifolius 3441, 3447
T. lamottei
Podospermum jacquinianum
Lactuca sp.
Majores
Chromopappus
Hebecarpuss
Collini
Profundisulcati
Angustissimi
Tragopogon
Brevirostres
Tragopogon + Brevirostres
T. porrifolius + T. lamottei
Out-groups
0.82
0.97
0.75
0.88
0.95
0.94
0.98
0.86
0.97
0.95
0.74
0.79
0.93
0.71
0.79
0.79
0.98
0.8
0.85
0.78
0.84
1.0
0.80
0.76
0.86
0.87
0.96
0.81
0.87
0.74
