# Supplementary material for: 250 years of hybridization between two biennial herb species without speciation
Source: AoB Plants. 2015 Jul 17;7:plv081. doi: 10.1093/aobpla/plv081 (PMC4571729; doi:10.1093/aobpla/plv081)
Supplement: Additional Information [file supp_plv081_plv081supp_table1.docx]

| Sample # | Morphology | Location code | Source Location | Latitude | Longitude | Genome size (pg) | British Museum Herbarium accession number |
| --- | --- | --- | --- | --- | --- | --- | --- |
| 429 | T. porrifolius | hd | Hadleigh Down | 51.542625 | 0.566998 | 6.25 | BM001139300 |
| 434 | Hybrid | hd | Hadleigh Down | 51.542625 | 0.566998 | 5.69 | BM001139307 |
| 437 | Hybrid | hd | Hadleigh Down | 51.542625 | 0.566998 | 5.72 | BM001139296 |
| 444 | T. pratensis | hd | Hadleigh Down | 51.542625 | 0.566998 | 5.22 | BM001139306 |
| 620 | Hybrid | cc 1 | Cooling Common | 51.436205 | 0.507516 | 5.75 | BM001139301 |
| 622 | Hybrid | cc 1 | Cooling Common | 51.436205 | 0.507516 | 5.73 | BM001139305 |
| 634 | Hybrid | cc 1 | Cooling Common | 51.436205 | 0.507516 | 5.83 | BM001139302 |
| 640 | T. porrifolius | dl | Decoys Lane | 51.474827 | 0.56924 | 6.21 | BM001139303 |
| 643 | T. porrifolius | dl | Decoys Lane | 51.474827 | 0.56924 | 6.29 | BM001139299 |
| 805 | Hybrid | chc 1 | Cannon Hill Common | 51.399057 | -0.220795 | 5.69 | BM001139297 |
| 900 | T. pratensis | dl | Decoys Lane | 51.474827 | 0.56924 | 5.07 | BM001139298 |
| 1000 | Hybrid | chc 3 | Cannon Hill Common | 51.402829 | -0.221051 | 5.54 | BM001139304 |
